# The impact of ocean acidification on the byssal threads of the blue mussel (*Mytilus edulis*)

**DOI:** 10.1371/journal.pone.0205908

**Published:** 2018-10-18

**Authors:** Grant Dickey, Brian M. Preziosi, Charles T. Clark, Timothy J. Bowden

**Affiliations:** School of Food and Agriculture, Aquaculture Research Institute, University of Maine, Hitchner Hall, Orono, ME, United States of America; Evergreen State College, UNITED STATES

## Abstract

Blue mussel (*Mytilus edulis*) produce byssal threads to anchor themselves to the substrate. These threads are always exposed to the surrounding environmental conditions. Understanding how environmental pH affects these threads is crucial in understanding how climate change can affect mussels. This work examines three factors (load at failure, thread extensibility, and total thread counts) that indicate the performance of byssal threads as well as condition index to assess impacts on the physiological condition of mussels held in artificial seawater acidified by the addition of CO_2_. There was no significant variation between the control (~786 μatm CO_2_ / ~7.98 pH/ ~2805 μmol kg^-1^ total alkalinity) and acidified (~2555 μatm CO_2_ / ~7.47 pH/ ~2650 μmol kg^-1^ total alkalinity) treatment groups in any of these factors. The results of this study suggest that ocean acidification by CO_2_ addition has no significant effect on the quality and performance of threads produced by *M*. *edulis*.

## Introduction

*Mytilus edulis*, is a sessile, intertidal species that is both economically and ecologically important to the coastline of the Gulf of Maine. The 2013–2017 commercial Maine landings for *M*. *edulis* were worth well over $2,000,000 [1] yet aquaculture accounts for less than 20% of U.S. mussel supply. In Maine, aquaculture accounts for an estimated 9% of total mussel supply [2]. Mussels can be grown from spat to market size using a range of methods including; suspended culture on hanging ropes and bottom culture in large beds. Successful cultivation of this species is dependent on the strength of the byssal threads *M*. *edulis* produces to anchor itself to hard substrates in the face of intense wave action present in the natural environment [3].

Byssal threads are made up of three regions: proximal, distal, and the plaque, which have slightly different chemical compositions [4]. To produce a thread, the mussel presses its foot against a solid substrate to create a chamber sealed off from the surrounding seawater and raises the roof of the chamber to create a negative pressure [5, 6]. The mussel then lowers the pH (2.0–6.0) and ionic strength of the water in the chamber before excreting the byssal proteins in liquid form from the tip of the foot [7, 8]. Exposure to seawater solidifies the plaque and thread [5, 6, 9]. The production of a single thread can take anywhere between 30 seconds and 8 minutes [8]. The threads start to lose their strength after about 25 days and must regularly be replaced with new ones [10]. Each mussel will maintain between 20 and 60 threads depending on the environmental conditions and time of year [4]. The strength of and/or production of byssal threads is dependent on many factors, including temperature, reproductive status [11, 12], salinity, tidal fluctuation, agitation [13], water velocity [14], and body size [15].

Open ocean systems are expected to experience a pH decrease of approximately 0.2 units by the year 2100, nearshore environments are expected to be more significantly impacted [16]. Due to coastal upwelling and fresh water input, coastal environments already experience lower and more variable pH than open ocean systems [17, 18]. As a resident of the near shore environment, *M*. *edulis* are expected to be exposed to lower and more variable pH. Previous studies have shown that the byssal thread strength of some *Mytilid* species can be negatively impacted by decreasing pH. Studies on *Mytilus trossulus* indicated that plaque strength decreases exponentially as pH decreases [19]. These species, however, have slight differences in the makeup of their byssal threads depending on the habitats and environmental factors they face [20].

The goal of this work was to examine how the changes in near-shore carbonate chemistry will affect the strength, functionality and number of byssal threads produced by the blue mussel *M*. *edulis*. The hypothesis that a CO_2_-induced decrease in pH will reduce the thread strength as well as the number of threads produced was tested. The load at failure, total extensibility and total thread counts were measured. These are important factors in assessing the quality and potential performance of the byssal threads being produced. In addition, the dry flesh weight and shell length were measured to provide a condition index for assessing the overall physiological condition of the mussels.

## Methods

### Acclimation/Sampling

In March 2017, approximately 100 mechanically debyssed mussels between 60 and 70 mm in shell length were supplied by Hollander & de Koning mussel farm located in Trenton, Maine. These mussels were transported to the Aquaculture Research Center at the University of Maine, Orono and separated into four identical systems. Each system is comprised of 4 specimen tanks (75 liter capacity per tank) along with a header tank and sump, for a total capacity of 632 liters per system. Artificial seawater made with Crystal Sea Marinemix salt (Marine Enterprises International, Baltimore, USA) was used for all the systems. The control systems were held at an average temperature of 10.2 ± 0.4°C (± s.d) and an average pH of 7.98 ± 0.10. This pH is well within the range of what *M*. *edulis* experience 70 miles south of the collection site in the Damariscotta estuary, which stays at or below 8.0 for months at a time (University of Maine EPSCoR SEANET data: http://maine.loboviz.com). The acidified systems were held at an average temperature of 10.2 ± 0.4⁰C and a pH of 7.47 ± 0.12. A pH of approximately 7.5 was chosen for the acidified system because it matches the parameters set forth in O’Donnell et al. which this study is designed to replicate. The CO_2_ was maintained using a Pentair Point Four RIU3 remote monitor/controller connected to a solenoid valve and a canister of beverage grade CO_2_. Each tank was lined with a 2-inch layer of pebbles measuring between 2 and 10 mm in diameter. The pH and temperature were monitored using Honeywell Durafet pH probes (model # 07777DVP-01-01) and were recorded twice daily [21]. Dissolved oxygen and atmospheric pressure were measured (ProODO professional series, YSI, Yellow Springs, USA) twice daily. Total alkalinity was measured daily using a benchtop colorimeter (Lamotte smart3 colorimeter) and weekly through titration (Table 1). Titrations were performed using the open cell titration method [22] with a 5 ml sample volume and a 0.01 N HCL acid solution in 32 ppt NaCl solution. In accordance with previous studies, increases in alkalinity associated with the use of artificial seawater were addressed by elevating the amount of CO_2_ being added into the acidified system to obtain a pH that was low enough to compensate for the increased alkalinity [23]. CO_2_ partial pressure was monitored daily in the header space of each tank using LiCor (LI-840A) sensors. However, the pCO_2_ in the water was calculated using the program CO2SYS (ver. 2.1 http://cdiac.ess-dive.lbl.gov/ftp/co2sys/) (Table 1). A 10% water change was conducted on all systems once a week.

**Table 1:** Environmental parameters of the treatments.

Treatment	Salinity	Temp (˚C)	pH (NBS Scale)	Total Alkalinity (μmol kg^-1^)	PCO_2_ μatm	ΩAr	ΩCa	Dissolved oxygen	Pressure (atm)
Control	34	10.2 ± 0.4	7.98 ± 0.10	2805 ±102	786	1.7	2.7	10.16	0.996
Acidified	33	10.2 ± 0.4	7.47 ± 0.12	2679 ± 95	2607	0.5	0.8	10.06	0.996

The dissolved oxygen of all tanks was always kept above 9.0 mg/L through constant aeration in the sump. PCO_2_, ΩAr, ΩCa were calculated for each tank in CO2SYS using the temperature, salinity, pH and total alkalinity values. Temp and pH are averages ± s.d. of the daily measurements taken by Durafet electrodes. Dissolved oxygen and pressure are averages of daily data. Water was undersaturated with respect to both aragonite and calcite in the acidified treatment. n = 116 (total number of measurements taken from each tank).

Algae mix (Shellfish Diet 1800, Reed Mariculture, Campbell, California, USA) was administered at a rate of 5% dry tissue weight per day [19]. To remove buffer components affecting alkalinity, the feed was diluted 1:4 in distilled water and centrifuged for 30 minutes at 7000 xg’s and 10 ⁰C. The algal pellet was then resuspended in distilled water. Each tank was fed 15 ml of resuspended feed (at a concentration of 5 x 10^8^ cells per ml) in the morning and 30 ml in the afternoon to maintain the optimal algae cell count in the system (<800 cells/μl) [24]. Cell counts were performed using a hemocytometer to determine residence time of algal cells in the system and the feeding regime was adjusted to ensure that feed was added only once the concentration of cells was reduced to 1.0 x 10^5^ cells in the system. This ensured that excess algae mix did not build up in the system and inhibit feeding due to high concentrations.

The mussels were placed in the tank and allowed to attach to the substrate for 16 days. At day 16 unattached mussels were removed and discarded. Threads of attached mussels were cut close to the shell margin. The mussels were maintained (4 animals per tank) for a further 38 days before their byssal threads were trimmed at the shell once again. They were then given 3 days to produce new threads. Threads attached to the gravel substrate were counted and then excised from each mussel at the byssal gland [4]. Threads attached to the tank (as opposed to the gravel substrate) were also counted but not included in the strength or extensibility tests. Mantle tissue samples (less than 20 milligrams per mussel) were taken and stored in 95% ethanol for later DNA analysis. The flesh was removed from the shell for later use in calculation of the condition index. The byssal threads were air dried in a cold room until tested [25].

### Byssal strength testing

One day prior to testing, the dried byssal thread clumps were fastened, by the byssal gland, between corrugated plastic using cyanoacrylate and threads were detached from the gland until there was one thread left from each animal tested. The threads were rehydrated in 35 ppt artificial seawater, at 10˚C, then placed in a 35 ppt artificial seawater bath, at 10˚C in an Instron tensile tester (Model 8801, Instron, Norwood, MA, USA), fitted with a 10–10,000 N load cell. Threads were extended at a speed of 10 mm/min until failure [4].

Breaking strength was measured as the last load value recorded by the Instron before the thread failed. Extensibility (a unitless measure) was measured as the length of the thread at time of failure divided by the original length of the thread [12]. Threads that became unglued at the corrugated plastic (n = 8) were not included in the final data analysis.

### Species verification

Less than 20 milligrams of wet mantle tissue was taken from each mussel and DNA was extracted using a modified-Chelex method [26]. The DNA samples were amplified by PCR using *M*. *edulis* specific primers (forward—GTAGGAACAAAGCATGAACCA and reverse GGGGGGATAAGTTTTCTTAGG) [27]. The PCR reaction mix utilized SSO advanced universal SYBR green supermix (Biorad) and standard conditions (94˚C for 3 minutes, followed by 30 cycles of 94˚C for 30 seconds, 54˚C for 30 seconds and 72˚C for 1 minute and a final elongation step of 72˚C for 1 minute). The products were resolved on 1.5% agarose gels and product size used to confirm species identification of the sample.

### Condition index

Data for a condition index was taken to assess if the acidified and control mussels were able to maintain a similar overall physiological state. At the end of the experiment, the length and weight of whole mussels were recorded. The flesh was then excised and dried at 60°C for at least 2 days until a constant weight was obtained. The condition index was calculated by dividing the dry flesh weight by the shell length cubed [19].

### Data analysis

All data analysis was performed using Graphpad Prism version 6.0. The data was tested on a group by group basis for normality (Shapiro-Wilk, p ≥ 0.1165) and equal variance (F test, p ≥ 0.2900). T-tests were run to check for significance differences in condition index, thread strength, thread extensibility and number of threads produced. Fisher’s exact test was used to test for a significant difference in thread failure location. All tests were run at the 0.05 significance level. A single thread from each tank (selected using random number generation) was selected for use in this analysis, resulting in 4 threads per system and 2 systems per treatment. One tank from the acidified group was excluded due to lack of sample threads.

## Results

### Survival and condition index

There were 9 mortalities during the 41-day acclimation period across the 2 treatment groups. These mortalities were spread across the two treatment groups. The survival rate was at least 85% for the mussels in both control and acidified treatments. There was no significant difference in the condition index (dry flesh weight/ shell length cubed) of the control and acidified group (t-test, p = 0.5408). The means were 0.00861 ± 0.00010 g cm^-3^ for the pH 8.0 mussels and 0.00755 ± 0.00141 g cm^-3^ for the pH 7.5 mussels (S1 Appendix).

### Thread counts

**Fig 1:**
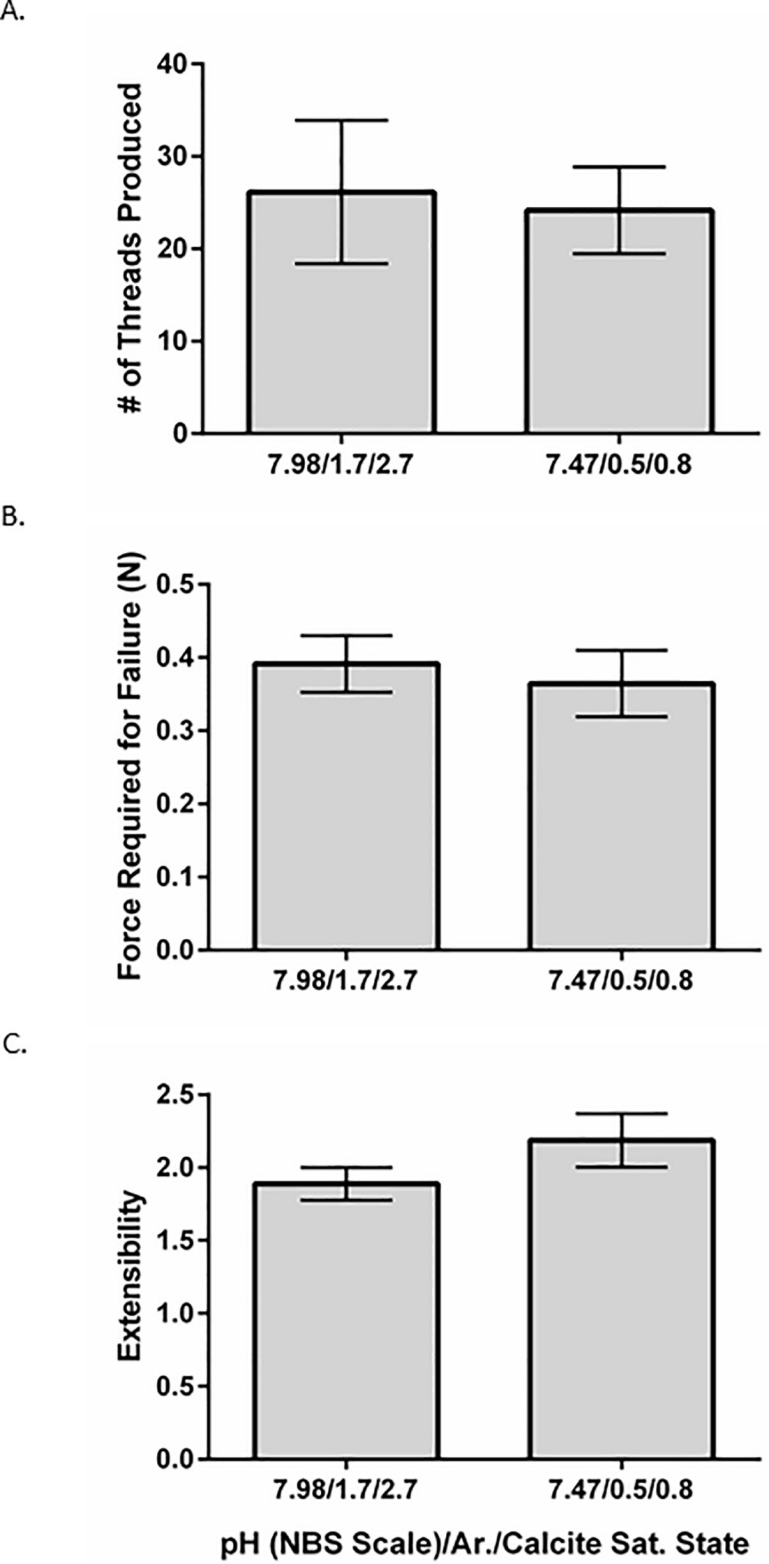
Byssal thread counts, force required for thread failure, and extensibility. Graphs showing the number of threads produced (A), force required to cause thread failure (B), and the extensibility of tested threads on the last day of the experiment (C) for the control group (on the left) and acidified group (on the right). Threads were counted 3 days after they were cut at the shell margin. Extensibility was determined by dividing the thread length at the time of failure by the length before stretching. Control group n = 8 while the acidified group n = 7. Error bars indicate ± standard error of the mean. See S1 Appendix for source data.

There was no significant difference between total number of threads produced by the two treatment groups. (t-test, p = 0.8362). The means were 26 ± 22 threads for the pH 8.0 mussels and 24 ± 12 threads for the pH 7.5 mussels (Fig 1A).

### Thread strength and failure location

There was no difference in thread strength between control mussels (pH 8.0) and acidified mussels (pH 7.5) (t-test, p = 0.6557). The mussels held at a pH of 8.0 had an average breaking load of 0.39 ± 0.11 N while the mussels held at a pH of 7.5 had an average breaking load of 0.36 ± 0.12 N (Fig 1B). There were 33% more breaks at the plaque region in the acidified tanks compared to the control tanks, but this difference was not significant (Fisher’s exact test, p = 0.2000) (S1 Appendix).

### Thread extensibility

There was no difference in thread extensibility between the control and acidified groups (t-test, p = 0.1769). Control animals averaged 1.9 ± 0.3 and acidified animals averaged 2.2 ± 0.5 (Fig 1C).

### Species verification

All PCR samples exhibited bands at between 350 and 380 bp, consistent with the sizes expected from *M*. *edulis*.

## Discussion

There was no significant difference in condition index, load at break, thread counts, and extensibility between the mussels kept at a pH of 8 and those kept at a pH of 7.5. These results indicate that byssal threads in *M*. *edulis* are not significantly impacted by an increase in ocean acidification.

O’Donnell et al. found that byssal threads were significantly weaker, as well as significantly less extensible when formed in and exposed to water with ≥1,200 μatm pCO_2_ (~7.60 pH) for 20 days [19]. They reported threads exposed to a pH of 7.79 or greater had average breaking strengths between 0.25 and 0.4 N [19]. The breaking strengths found for the control and acidified groups in the current study are also within this range. Zhao et al. found that the force required to break individual threads was reduced by 26%, 41.8%, and 23.9% after a week exposure to 1,207 μatm pCO_2_ (~7.80 pH), 1976 μatm pCO_2_ (~7.60 pH), and 3140 μatm pCO_2_ (~7.40 pH) treatments respectively. They also reported a reduction in thread production by 45.9%, 31.1%, 56.3% for the above treatments respectively [28]. It should be noted that the strength tests performed by Zhao et al. were performed on dry threads at room temperature while most prior studies tested the threads in seawater [4, 11, 19]. It is unclear if this difference in methodology had an impact on reported thread strengths.

The goal of the current study was to examine the chronic effects of acidification on mussel threads. It is possible but not likely that the acidified group adapted to the CO_2_ conditions over the course of the experiment. The lack of any significant differences between condition index in control and acidified groups indicates that the animals in the acidified conditions did not use any more major physiological resources than the control group during the experiment to mount an acute response (S1 Appendix). O’Donnell et al. also found no difference in condition index among any of their treatments for *M*. *trossulus* even though they did see an effect on thread strength [19]. This suggests that *M*. *trossulus* (which lives in the same type of environment as *M*. *edulis*) is not capable of short-term adaptation to CO_2_ during an acclimation period that was similar to the one used in the current study [19, 29]. Current oceanographic data indicates *M*. *edulis* are exposed to variable pH conditions in their natural environment (University of Maine EPSCoR SEANET data: http://maine.loboviz.com). This means *M*. *edulis* either mounts acute responses to low pH environments in quick succession or is already adapted to deal with the stressor on a chronic basis. Even if the results described in the current study are due to the mussels having an acute response to this increase in CO_2,_ it is evident that these organisms are fully equipped to deal with an increase in ocean acidity and will be unaffected by an acidity increase of this magnitude in the natural environment.

The mussels used in the current work, as well as those in O’Donnell et al. and Zhao et al., were fed once if not twice a day to make food constantly available to the mussels [19, 28]. Previous work shows that juvenile mussels produce weaker threads when starved [30] and that an adequate food supply can prevent the detrimental effects of moderate acidification on shell calcification and growth [31]. If an abundance of food allows *M*. *edulis* to produce stronger threads, it is possible *M*. *edulis* will be weakened by ocean acidification during times of natural food shortages. Given that mussel aquaculture operations are generally done out in the open environment (where food concentrations cannot be controlled) it may be worthwhile for future studies to test if food supply has an impact on thread strength in relation to acidification.

O’Donnell et al. and Zhao et al. both observed an increase in breaks at the plaque, and a decrease in breaks in the distal region, when pH decreases [19, 28]. Analysis from the current study showed a 33% increase in breaks in the plaque region, but this increase was not significant (S1 Appendix). Further study of *M*. *edulis* threads at lower pH values than those tested here may help establish a pH that causes weakened plaques. This would be useful information for growers in the case of short-term extreme acidification events resulting from sudden freshwater input during storms [18].

This study examines a different species than previous studies which likely has a significant impact on the differences in findings across said studies. Previous work characterized 95% of the mussels in the sampling area to be *M*. *edulis* [32]. Present data verified that all experimental animals were *M*. *edulis* [27]. It is possible that *M*. *edulis* is accustomed to a larger range in pH than *M*. *coruscus*, making it difficult to generalize the findings on *M*. *edulis* to this species. *M*. *coruscus* lives in a relatively warm environment and likely exhibits mechanical differences in the threads when compared to other *Mytilus* species [25]. This may explain why the required breaking force for the control group threads of *M*. *coruscus* (~1.5N) is about 4 times those recorded for *M*. *edulis and M*. *trossulus* (~0.35N) [19, 28] as well as why observed extensibility is higher in *M*. *edulis*. On the other hand, *M*. *trossulus* has both a similar thread structure to that of *M*. *edulis* [4] and a similar force needed to break threads produced at an ambient pH [19]. There may still be differences in conditions the threads are capable of solidifying in, as mussels are capable of altering the expression of genes that encode for proteins used to make the threads [28].

The effect of ocean acidification on *M*. *edulis* byssal threads is crucial to the mussel growers and natural beds in the Gulf of Maine as well as up and down the east coast of the U.S. While our results show there is no cause for concern right now, further study is required to determine what affect increasing temperatures occurring alongside ocean acidification will have on the threads of this species as increased effects of ocean acidification could be catastrophic for aquacultured as well as natural bed mussels in the future. These studies should address if further acidification of seawater will affect the strength and extensibility of the fibers and whether a shift in breakage location is an early indicator of damage caused by decreasing pH.

## Supporting information

S1 Appendix (DOCX)
