# Supplementary material for: The impact of ocean acidification on the byssal threads of the blue mussel (Mytilus edulis)
Source: PLoS One. 2018 Oct 18;13(10):e0205908. doi: 10.1371/journal.pone.0205908 (PMC6193707; doi:10.1371/journal.pone.0205908)
Supplement: S1 Appendix — (DOCX) [file pone.0205908.s001.docx]

Control Treatment Thread Data (7.98 ± 0.10 pH, NBS Scale)

| Tank # | Initial Thread Length (mm) | Length Stretched (mm) | Length at Thread Failure (mm) | Extensib. | Load at break (N) | Break Location | Thread Count |
| --- | --- | --- | --- | --- | --- | --- | --- |
| 1 | 19 | 27 | 46 | 2.4 | 0.40 | plaque | 57 |
| 2 | 16 | 7 | 23 | 1.4 | 0.21 | plaque | 23 |
| 3 | 15 | 10 | 25 | 1.7 | 0.35 | distal | 61 |
| 4 | 27 | 26 | 53 | 2.0 | 0.43 | plaque | 2 |
| 13 | 41 | 29 | 70 | 1.7 | 0.51 | distal | 8 |
| 14 | 18 | 13 | 31 | 1.7 | 0.55 | distal | 24 |
| 15 | 14 | 16 | 30 | 2.1 | 0.37 | plaque | 24 |
| 16 | 18 | 19 | 37 | 2.1 | 0.31 | plaque | 10 |

Acidified Treatment Thread Data (7.47 ± 0.12 pH, NBS Scale)

| Tank # | Initial Thread Length (mm) | Length Stretched (mm) | Length at Thread Failure (mm) | Extensib. | Load at break (N) | Break Location | Thread Count |
| --- | --- | --- | --- | --- | --- | --- | --- |
| 5 | 21 | 19 | 40 | 1.9 | 0.19 | plaque | 22 |
| 6 | 12 | 15 | 27 | 2.3 | 0.36 | plaque | 28 |
| 7 | 20 | 21 | 41 | 2.1 | 0.33 | plaque | 46 |
| 9 | 13 | 25 | 38 | 2.9 | 0.58 | plaque | 13 |
| 10 | 24 | 39 | 63 | 2.6 | 0.42 | plaque | 33 |
| 11 | 27 | 12 | 39 | 1.4 | 0.38 | plaque | 14 |
| 12 | 12 | 13 | 25 | 2.1 | 0.29 | plaque | 13 |

**Mussel Thread Data.**

The tables above list the all thread-related data for the mussels used in the final data analysis and construction of Figure 1. Each row represents data from a single animal. No more than one animal per tank was used for data analysis. “Tank #” indicates which tank the mussel was kept in. Tanks 1-4 and 13-16 were part of the control treatment while tanks 5-8 and 9-12 were part of the acidified treatment. No animal was sampled from Tank 8 due to breakage of sample threads prior to testing. The “Extensib.” column indicates extensibility, which was calculated by dividing the thread length at the time of failure by the intial thread length. “Load at Break” indicates the force reading given by the tensometer at the moment of thread failure. The “Break Location” indicates which portion of the thread failure occurred at. “Thread Count” indicates how many threads were present and attached to substrate after the 3 day regrow period at the end of the experiment.

Control Treatment Condition Index Data (7.98 ± 0.10 pH, NBS Scale)

| Tank # | Length of Shell (cm) | Dry Flesh Weight (g) | Condition Index (g/ cm^-3^) |
| --- | --- | --- | --- |
| 1 | 6.41 | 1.88 | 0.00715 |
| 2 | 6.52 | 2.29 | 0.00827 |
| 3 | 7.29 | 4.84 | 0.01249 |
| 4 | 6.27 | 1.06 | 0.00429 |
| 13 | 7.52 | 3.98 | 0.00936 |
| 14 | 6.94 | 2.45 | 0.00732 |
| 15 | 5.44 | 1.19 | 0.00741 |
| 16 | 5.34 | 1.92 | 0.01262 |

Acidified Treatment Condition Index Data (7.47 ± 0.12 pH, NBS Scale)

| Tank # | Length of Shell (cm) | Dry Flesh Weight (g) | Condition Index (g/ cm^-3^) |
| --- | --- | --- | --- |
| 5 | 5.62 | 2.41 | 0.01358 |
| 6 | 6.90 | 2.26 | 0.00689 |
| 7 | 6.29 | 2.96 | 0.01190 |
| 9 | 5.02 | 0.71 | 0.00564 |
| 10 | 6.69 | 1.85 | 0.00618 |
| 11 | 5.23 | 0.51 | 0.00355 |
| 12 | 5.98 | 1.10 | 0.00513 |

**Mussel Condition Index Data.**

The tables above list the all the condition index data for the mussels used in the final data analysis as well as length of the shells. The condition index was calculated by dividing the dry flesh weight by the shell length cubed. Each row represents data from a single animal. No more than one animal per tank was used for data analysis. “Tank #” indicates which tank the mussel was kept in. Tanks 1-4 and 13-16 were part of the control treatment while tanks 5-8 and 9-12 were part of the acidified treatment. All condition index data was taken at the end of the experiment.
